# Perspectives of older adults with chronic illness on person-centered practice at an inpatient hospital department: a descriptive study

**DOI:** 10.1186/s12877-024-05261-1

**Published:** 2024-08-29

**Authors:** Diana Vareta, Filipa Ventura, Carlos Família, Célia Oliveira

**Affiliations:** 1 PhD Program, University of Lisbon (UL) and Nursing School of Lisbon (ESEL), Lisboa 1600-214, Portugal; 2 Egas Moniz Interdisciplinary Research Centre (CiiEM), Egas Moniz Universitary Institute, Quinta da Granja, Monte de Caparica 2829-511, Portugal; 3 The Health Sciences Research Unit: Nursing (UICISA:E), Nursing School of Coimbra (ESEnfC), Coimbra 3000-076, Portugal; 4 Laboratory of Molecular Pathology and Forensic Biochemistry, Egas Moniz Universitary Institute, Quinta da Granja, Monte de Caparica 2829-511, Portugal; 5 Nursing School of Lisbon (ESEL), Lisboa 1600-096, Portugal

**Keywords:** Patient-centred care, Person-centered practice inventory – care, Aged, Inpatient, Noncommunicable diseases

## Abstract

**Background:**

The growing aging trend associated with a higher prevalence of chronic illnesses is increasing the demand for the development of person-centered practice in specific care settings. Knowing the person’s perception of the care and the care experience is essential to improving inpatient care toward person-centeredness. This study aims to characterize the perceptions of person-centered practice of hospitalized older adults with chronic illness at a Portuguese inpatient hospital department.

**Methods:**

A quantitative, descriptive, cross-sectional approach was followed. Data were collected using a sociodemographic and health history questionnaire and the Person-Centered Practice Inventory - Care (PCPI-C). The effect of the different variables on each PCPI-C construct was determined using analysis of variance (ANOVA).

**Results:**

The results show that person-centered practice was positively perceived in the five constructs of the *person-centered processes* domain (M = 3.92; SD = 0.47). The highest-scored construct was *working with the person’s beliefs and values* (M = 4.12; SD = 0.51), and the lowest was *working holistically* (M = 3.68; SD = 0.70). No significant effect of the independent variables was found to influence the perceptions of any of the constructs in the *person-centered processes* domain.

**Conclusions:**

These results might indicate that person-centered processes are perceived uniquely by each person through individualized therapeutic relationships rather than a pattern of care shared by hospitalized older adults.

**Supplementary Information:**

The online version contains supplementary material available at 10.1186/s12877-024-05261-1.

## Background

In contemporary healthcare, the concept of person-centered care has emerged worldwide as a transformative approach, redefining the fundamental focus of healthcare in care settings [1, 2]. Person-centered care represents a paradigm shift from the traditional healthcare delivery model, which focuses primarily on the medical condition rather than the person being cared for [3, 4]. This approach integrates the perspectives of individuals, families, and communities as users and participants, recognizing the person’s unique identity as an integral part of the care process. It is described as “an approach to practice that is established through the formation and fostering of therapeutic relationships between all care providers, service users, and others significant to them, underpinned by values of respect for persons, individual right to self-determination, mutual respect, and understanding” (p. 5) [5].

Person-centered practice (PCP) can generate significant benefits to the health and healthcare of all people throughout their lifespan, especially for those with complex care needs affecting their daily lives [6–8]. The growing aging trend is leading to an increased demand for person-centred care processes, as a proportion of the older population experiences biological, social, psychological, and cognitive frailties, increasing functional limitations, levels of dependency, and difficulties in the ability to respond or adapt [9, 10]. Additionally, the aging process is associated with a higher prevalence of chronic illnesses, leading many older adults to use health services most frequently, search for hospital care recurrently, and look for a broader spectrum of care support, often involving several healthcare professionals [9, 11]. This fact, in turn, elevates the potential for fragmented care relationships and hospitalization-associated complications [9, 12–15].

When thinking about improving inpatient care toward person-centeredness, in addition to assessing the care provided by health professionals, it is essential to know the person’s perception of the care received and the care experience [16–18]. Only with that knowledge will it be possible to identify which aspects of care can be restructured to effectively meet the person’s needs and expectations. Few studies provide access to the perception of the older person with a chronic illness about PCP, particularly in the context of hospitalization [19–21].

The development of the Person-Centered Practice Framework (PCPF) [3, 5] and the instruments designed for assessing it in clinical practice [22–24] have contributed to its implementation in healthcare contexts. The PCPF outlines the essential domains and core concepts of person-centered care, providing guidelines for its application and development in practice [3, 5].

The PCPF consists of five domains: the macro context, which encompasses strategic and political factors influencing the development of person-centred cultures; prerequisites, which focus on the attributes of staff; the practice environment, which pertains to the context in which healthcare is delivered; person-centred processes, which involve methods of engagement necessary for creating connections between individuals; and the outcome, representing the result of an effective person-centred practice, a healthful culture [3]. The relationships between these domains indicate that strategic and policy considerations must first be addressed. Subsequently, staff attributes are prerequisites for managing the practice environment and engaging effectively through person-centred processes. This sequence ultimately leads to achieving a healthful culture, which is the central element of the framework. Additionally, it is essential to acknowledge relationships and overlaps among the constructs within each domain [25].

The Person-Centered Practice Inventory (PCPI) is an instrument aligned with the theoretical elements of the PCPF, offering an understanding of its practice, identifying areas of potential improvement, and designing specific interventions to elevate the operationalization of the PCP [22–24]. The PCPI-C is designed to evaluate service users’ experiences within the person-centred processes domain. This domain, which include working with the person’s beliefs and values, sharing decision-making, engaging authentically, being sympathetically present, and working holistically, is the component of care that directly impact service users’ experiences [23].

This study is part of a clinical study protocol [26] designed to provide recommendations for improving the PCP in the daily care of hospitalized older adults with chronic illness at an internal medicine department. The current practice analysis refers to how the PCP is perceived and identified in the context under study from the eyes of hospitalized older adults, considering that all principles and domains presented in the PCPF are fundamental for implementing this practice. Therefore, the present study aims to characterize the perceptions of hospitalized older adults with chronic illnesses about PCP. The absence of published studies establishing the relation between patients’ individual and health characteristics and their perception of PCP prompts us to explore the influence of sociodemographic and health history variables on their perceptions.

## Methods

### Design and population

The study followed a quantitative, descriptive, cross-sectional approach. It was conducted at an internal medicine inpatient unit of a secondary hospital in an urban area of Portugal. The hospital has a direct influence area of 500,000 inhabitants, and the internal medicine unit comprises 55 inpatient beds. All the inpatients at this unit were eligible to participate in the study if they met the inclusion criteria during the data collection period. A sample of 200 inpatients was calculated to guarantee the statistical validity of the results.

### Inclusion criteria

Older adults were eligible to participate in the study if they were over 65 years old, had a chronic disease diagnosis, were hospitalized in the study setting (i.e., the inpatient internal medicine department) for more than 48 h, could understand, read and communicate in Portuguese, and wished to participate.

### Exclusion criteria

The exclusion criterion was the presence of cognitive impairment, as assessed by the 6-Item Cognitive Impairment Test (6-CIT) [27]. In addition, inpatients considered clinically frail to participate by the recruiting nurse were ineligible to participate in the study.

### Data collection

Data were collected between February and June 2023. The nurse in charge at the internal medicine department’s identified the eligible participants according to the inclusion and exclusion criteria, then the researcher applied 6-CIT, the informed consent form and provided a questionnaire including sociodemographic and health history characterization and the Person-Centered Practice Inventory - Care (PCPI-C).

The PCPI-C is a psychometrically accepted and validated scale by an international panel of experts in the PCP with proven reliability [23]. It allows us to understand the perception of person-centeredness with the care experience from the eyes of the person. The PCPI-C is a self-reported instrument consisting of 18 items on a 5-point Likert-type response scale, where higher scores indicate better agreement. The PCPI-C measures the *person-centered processes* domain derived from the PCPF and comprises five constructs: *working with the person’s beliefs and values*,* sharing decision-making*,* engaging authentically*,* being sympathetically present*, and *working holistically* [23]. The *person-centered processes* domain describes PCP in context, focusing specifically on the care provided to the person through a series of activities [3]. The constructs are often interlinked in care provision and are synergistic because their interaction magnifies the results [3].

The PCPI-C has been translated and culturally adapted into Portuguese with acceptable psychometric properties and good reliability [28]. A questionnaire was developed to characterize the sample’s sociodemographics, including sex, age, living environment, residence, home care, educational level, and health history, comprising actual diagnosis, length of hospital stay, previous hospitalizations, number of prior hospitalization episodes, health history, and level of dependency with the Barthel index score [29, 30]. The inventory and the questionnaire were made available to the participants for self-reporting in paper format and is expected to take around 18 min to complete. In case of difficulties in self-completion by the participants, assistance was provided. In these cases, the principal investigator conducted the interview strictly following the PCPI-C questions, ensuring the confidentiality of the information collected and without adding any elements that would allow the participants to be identified. The data were collected as close to discharge from the department as possible to ensure sufficient knowledge of the subject under study and the stabilisation of the clinical situation. The data was transferred to Google Forms^®^ by the researcher, and the database was re-checked to identify discrepancies.

### Statistical data analysis

G*Power v.3.1 was used to compute the sample size [31]. One-way ANOVA was chosen because the main objective was to compare the differences in the PCPI constructs between groups defined by sociodemographic characteristics and health history. A moderate effect size of 0.3 and an α of 0.05 were used for eight groups. A total sample size of 168 was required to achieve a power of 0.80. Considering the possibility of a drop-out rate of approximately 20%, the total sample size was defined as 200 persons.

The statistical package for social sciences software (IBM SPSS Statistics^®^ for Windows, v.29.0. IBM Corp. Released 2023, Armonk, NY, USA) was used to analyze the quantitative data and produce all plots. Missing data analysis revealed no missing data for the 18 items. A descriptive analysis (i.e., mean, standard deviation, minimum, and maximum) of the PCPI-C constructs was performed. Subsequently, analysis of variance (ANOVA) was conducted to determine the effect of demographic and health history on the PCPI-C constructs described in the previous section. In addition, Tukey’s post-hoc test for multiple comparisons was used to evaluate dependent variables with more than two response options, for which statistically significant differences were found in ANOVA. Analysis of the Q-Q plot of the residuals and the histogram of the residuals were used to assess the model assumptions. A p-value < 0.05 was considered for statistical significance [22, 32], and values were rounded to the nearest hundredth.

### Ethical considerations

Ethical approval was obtained before study execution from the Hospital’s Ethics Committee, where the study took place (ref. nr. 36/2021). All procedures were performed following the Declaration of Helsinki [33] and in compliance with the General Data Protection Regulation [34]. Permission to use the PCPI-C and 6-CIT was granted upon request to the respective authors.

Prospective participants were first given oral and written information about the study, including its purpose, relevance, data collection methods, expected participation, and disclosure of the information collected. Between the presentation and validation of understanding of the information provided and the start of data collection, a reflection period of at least 24 h was ensured so that participants could consider their decision and complete the informed consent form.

## Results

### Characteristics of the participants

The sample included 192 inpatients, excluding those who did not complete the PCPI-C thoroughly (4%). All the participants were Portuguese and between 65 and 91 years old (M = 75.05; SD = 7.37). The gender distribution was similar, with a slight predominance of females (54.2%) over males. The educational level ranged between no academic education (16.7%) and graduate (3.6%), with a predominance of people with a middle school education (39.6%) (Table 1).

Concerning residence, most participants lived primarily in urban areas (54.2%), in their own homes, or in relatives’ homes (78.6%). Among the participants living at home, 46.9% had no domiciliary social or healthcare support, 26.6% had assistance from healthcare professionals, and 26.6% received care from informal carers (Table 1). Only 21.4% of the participants lived in a public or private residential facility that provides a high level of long-term personal or nursing care for persons.

Previous hospitalization experience was identified by 75% of participants, and 53.6% of those had between 1 and 3 inpatient episodes (Table 2).

In the current hospitalization experience, the older adults stayed on average seven days (M = 7.17; SD = 6.93; Min = 2; Max = 93), and the most frequent diagnosis were diseases of the circulatory system (41.7%), followed by diseases of the respiratory system (24%), diseases of the genitourinary system (9.9%), metabolic, endocrine, and nutritional diseases (6.3%) and hematological and autoimmune diseases (4.7%). Other diagnoses were presented by 13.5% of the participants (Table 1). Regarding the level of dependence during hospitalization, the Barthel index showed that almost half of the population had severe dependence (48.4%), followed by moderate dependence (37%), complete independence (8.3%), and total dependence (6.3%) (Table 2).

The most prevalent health history characteristic in the study sample concerned the circulatory system (33.3%), followed by metabolic, endocrine, and nutritional diseases (19.3%), oncological disease (14.1%), and respiratory system (9.9%) (Table 2).

### Perception of person-centered practice

The results were analyzed using the mean score of the response scale (1 to 5 points), according to the authors’ guidelines [23]. Constructs with a mean score higher than 2.5 were considered positive, indicating agreement among inpatients.

The results show that *the person-centered processes* domain had a high score (M = 3.92; SD = 0.47), and all its constructs were positively scored (Table 3). Two constructs with very high scores emerged, namely *working with the person’s beliefs and values* (M = 4.12; SD = 0.51) and *being sympathetically present* (M = 4.06; SD = 0.53). *Working holistically* (M = 3.68; SD = 0.70) and *sharing decision-making* (M = 3.78; SD = 0.60) had the lowest scores (Table 3).

**Table 1:** Sociodemographic characteristics of the participants

	*N* = 192	
**Sex**		
Female	104 (54.2%)	
Male	88 (45.8%)	
**Age**		
[65, 69]	57 (29.7%)	
[70, 74]	36 (18.8%)	
[75, 79]	46 (24%)	
[80, 84]	28 (14.6%)	
[85, 89]	17 (8.9%)	
≥ 90	8 (4.2%)	
**Living environment**		
Rural	88 (45.8%)	
Urban	104 (54.2%)	
**Residence**		
Home	151 (78.6%)	
Nursing home	41 (21.4%)	
**Home care**		
Health professional	51 (26.6%)	
Informal carer	51 (26.6%)	
None	90 (46.9%)	
**Educational level**		
Graduate	7 (3.6%)	
High school	10 (5.2%)	
Middle school	76 (39.6%)	
Elementary school	67 (34.9%)	
Uneducated	32 (16.7%)	

**Table 2:** Health history characteristics of the participants

	*N* = 192	
**Previous hospitalization**		
No	48 (25%)	
Yes	144 (75%)	
**Number of previous episodes**		
0	48 (25%)	
[1, 3]	103 (53.6%)	
[4, 6]	34 (17.7%)	
≥ 7	7 (3.6%)	
**Health history**		
Diseases of the circulatory system	64 (33.3%)	
Diseases of the genitourinary system	10 (5.2%)	
Diseases of the nervous system	13 (6.8%)	
Diseases of the respiratory system	19 (9.9%)	
Hematological and autoimmune diseases	11 (5.7%)	
Metabolic, endocrine, and nutritional diseases	37 (19.3%)	
Oncological diseases	27 (14.1%)	
Other diseases	11 (5.7%)	
**Actual diagnosis**		
Diseases of the circulatory system	80 (41.7%)	
Diseases of the genitourinary system	19 (9.9%)	
Diseases of the respiratory system	46 (24%)	
Hematological and autoimmune diseases	9 (4.7%)	
Metabolic, endocrine, and nutritional diseases	12 (6.3%)	
Other diseases	26 (13.5%)	
**Length of stay**		
[2, 3]	6 (3.1%)	
[4, 6]	101 (52.6%)	
[7, 9]	71 (37.0%)	
≥ 10	14 (7.3%)	
**Barthel index**		
Complete independence	16 (8.3%)	
Moderate dependence	71 (37.0%)	
Severe dependence	93 (48.4%)	
Total dependence	12 (6.3%)	

**Table 3:** Mean and Cronbach’s alpha scores of the PCPF constructs

Constructs	Mean (SD)		Cronbach α	
*Person-centered processes*	3.92 (0.47)		0.88	
Working with the person’s beliefs and values	4.12 (0.51)		0.74	
Sharing decision-making	3.78 (0.60)		0.68	
Engaging authentically	3.98 (0.47)		0.58	
Being sympathetically present	4.06 (0.53)		0.65	
Working holistically	3.68 (0.70)		0.71	

### Influence of sociodemographic and professional characteristics in the person-centred processes

The *length of stay* was shown to have a significant influence on the perceptions of the construct of *sharing decision-making* (F(3) = 4.46, p-value = 0.005, partial η2 = 0.08) (Appendix A2). Furthermore, Tukey’s posthoc test for multiple comparisons revealed significant differences between the perceptions of those who spent 4–6 days (M = 3.84; SD = 0.06) and those who spent 7–9 days (M = 3.60; SD = 0.07) (t(3) = −0.23, *p* = 0.05) and also between those who spent 7–9 days (M = 3.60; SD = 0.07) and those who spent ≥ 10 days (M = 4.20; SD = 0.11) (t(3) = − 0.59, *p* = .003) (Appendix A.2).

Evaluation of the effect of the levels of independent variables, namely, sex, age, living environment, residence, home care, actual diagnosis, length of stay, number of previous episodes, health history, Barthel index score, and educational level, on the perceptions of the constructs *working with the person’s beliefs and values*, *engaging authentically*, and *being sympathetically present* showed no significant differences between groups (Appendix A). The *length of stay* was shown to significantly influence patients’ perception of the construct *working holistically*, though with a small effect size (F(3) = 5.17, p-value = 0.016, partial η2 = 0.06) (Appendix A5). However, no significant differences were identified with the subsequent multiple comparison tests, indicating that the observed variation may be due to random chance or other factors not captured in the posthoc analysis.

The effect of actual diagnosis on the perceptions of *sharing decision-making* construct, though not statistically significant, had a borderline p-value (F(5) = 2.05, p-value = 0.075, partial η2 = 0.06), showing that hematological and autoimmune diseases had a lower score (M = 3.22; SD = 0.68) than diseases of the respiratory system (M = 3.92; SD = 0.60) and autoimmune diseases (M = 3.22; SD = 0.68) than other diseases (M = 3.93; SD = 0.49) (Appendix A.2). However, it is essential to interpret this finding carefully due to the relatively small number of participants in the hematological and autoimmune diseases group (*n* = 12).

### Reliability of the PCPI-C

The inventory’s internal consistency was found adequate when applied to the study sample, with the *person-centered processes* domain presenting good consistency (α = 0.88). When assessing the internal consistency of each construct of the PCPI-C, good consistency was found when *working with the person’s beliefs and values* (α = 0.74) and *working holistically* (α = 0.71) [31]. All the constructs were significantly correlated (Appendix B).

## Discussion

In Portugal, demographic data reveal that 23.6% of the resident population is aged over 65, with 37.3% experiencing complete dependence and 71.4% living with a chronic illness [35]. These statistics underscore the importance of reorganizing healthcare services to address the older population’s needs. The sample reflects the reality of hospitalization in Portugal, with an average length of hospital stay of 7 days [36] and diseases of the circulatory and respiratory systems as the principal diagnosis for older adult in internal medicine departments [37].

Service users’ perceptions of PCP were positive, with all constructs having a mean score greater than 2.5 (Min = 3.68; Max = 4.11). *Person-centered processes* are the only domain of PCPF applied to capture the service user’s experiences of PCP. The constructs composing *person-centered processes* describe care delivery through a set of person-centered activities that directly impact service users’ experiences of care [3].

The construct *working with the person’s beliefs and values* had a higher score for the *person-centered processes* domain (M = 4.11; SD = 0.51), which demonstrates the service user’s recognition that health professionals explore and pay attention to their beliefs and values and how the person understands the current care experience. Getting acquainted with others’ values and beliefs demands genuinely caring about them and realizing their uniqueness as human beings through their perspective, psychosocial context, and social role. For healthcare professionals, the ability to work with a person’s beliefs and values forms the foundation of the PCP, as it determines engagement with practice and influences all the other constructs of *person-centered processes* [3].

The constructs of s*haring decision-making* (M = 3.78; SD = 0.60) and *working holistically* (M = 3.68; SD = 0.70) obtained lower scores in the domain and could be influenced by the length of stay of service users. However, having a shorter (2–3 days) or longer hospitalization (≥ 10 days) can increase the participants’ perception of these constructs. The reason may be that shorter stays can limit opportunities for decision-making involvement due to fast-paced treatment. In contrast, extended stays may facilitate more interactions with the healthcare team, positively affecting perceptions of decision-sharing and holistic care. Thus, considering the length of service users’ stay is crucial when interpreting these constructs, as it can shape their perceptions of care.

*Sharing decision-making* is related to facilitating involvement in the decision-making process by service users and their significant others, considering values, experiences, concerns, and future goals. Participation in decision-making requires providing information, empowerment, and a negotiation process considering the service user’s values, beliefs, and experiences. A cross-sectional study previously conducted in Portugal to assess patients’ preferred roles in healthcare-related decision-making [38] showed that most participants preferred a controlling role of the professional rather than actively participating in decision-making, especially among older people with fewer qualifications. The American Geriatrics Society Expert Panel on Person-Centered Care [39] states that healthcare professionals should include persons in the decision-making process to the extent they desire. However, the reasons for declining participation in decision-making should be identified [19]. Resistance may be related to a lack of energy related to health conditions, limited support from relatives, and familiarity with paternalistic-oriented healthcare systems, resulting in the indifference of older adults and the prevalence of expert opinions [7, 40–42]. Awareness of this fact is essential for healthcare professionals to further recognize the importance of involving the person in clinical decision-making through education and support to reverse the indifference trend and promote PCP [43].

*Working holistically* is a way of providing care that “collectively embraces the mind, body, and spirit of the person, in a culture of healthcare relationships that are collaborative and grounded in harmony and healing” (p. 155) [3]. The concept of holism has a longstanding presence in healthcare services. However, it depends on the healthcare professionals’ ability to integrate all the elements in their practice and the commitment to cultivate a caring culture that facilitates care aimed at the whole person. The lower score obtained for this construct, in comparison to that of the other constructs, might be related to the tendency of healthcare professionals to prioritize the disease and the diagnosis over understanding its impact on daily living from the person’s perspective. Allowing them to express their concerns and recognize how the disease influences their overall life circumstances [44]. This finding reveals the need for healthcare professionals to connect with the person through physiological, psychological, sociocultural, developmental, and spiritual dimensions. The ability to provide holistic care is strongly determined by how healthcare professionals engage with the person in the therapeutic relationship [3].

The construct of *engaging authentically* represents a dynamic approach to being genuinely in the therapeutic relationship. It is related to the person’s knowledge, professional clarity of beliefs and values, self-knowledge, and expertise. For a practitioner to authenticate with a person, the care situation should be approached as a unique interaction based on their values and beliefs and healthcare professional prerequisites, namely, their power-sharing capability [3]. Individuals should be encouraged to express their experiences, concerns, and ambitions openly, enabling healthcare professionals to engage authentically [43]. In the context of PCPF, this construct is directly linked with good care experience and healthful culture outcomes [3]. In this study, service users scored *engaging authentically* consensually (M = 3.98; SD = 0.47), recognizing its presence in the care they experience. *Being sympathetically present* had a similar score (M = 4.06; SD = 0.53) and describes a way of being with the person that recognizes the uniqueness and value of the individuals, identifies what is important to them in their life, and understands the feelings and experiences lived in the moment. To achieve this, healthcare professionals must be available to the person, which can be challenging in contexts with a heavy workload [5, 45].

All the constructs analyzed require a constant dynamic movement between the self and the context and the identification of which and how *person-centered processes* can be practiced in a unique relationship at a specific moment in time and place [3]. A comprehensive interpretation of the person’s perceptions implies analyzing the other domains that constitute the PCPF, namely the prerequisites and the care environment. These domains are determinants for adequate care and the operationalization of person-centered processes, contextualizing them in the macrocontext of health and recognizing their influence on care practice [5].

The independent variables did not significantly affect any of the constructs in the *person-centered processes* domain. A person’s perception, i.e., how they interpret the world around them, is highly subjective and influenced by several factors, such as life experience, education, cultural environment, social life, emotional state, motivations, values, and beliefs [46, 47]. In addition, each person has a set of qualities that characterize and distinguish them in the therapeutic relationship, which the authors of the PCPF refer to as personhood [5]. Personhood determines the way a person lives, how they relate to others, and how they perceive the situation they are currently experiencing.

Older inpatients adjust their expectations and perceptions of their healthcare experience to maintain balance through a particular and dynamic approach to regain a meaningful life after a destabilizing event such as hospitalization [46, 48]. Person-centered care is an individualized approach that values a person’s participation in the healthcare relationship, supports shared decision-making and mutual understanding, and respects a person’s values, preferences, and beliefs [3]. The absence of influence of independent variables on the *person-centered process* domain might reveal no tendency toward a pattern of care shared by the study sample but rather a finding of individualized care that has been perceived uniquely by each person. The care experience differs regardless of sociodemographic and health history characteristics. This result reinforces the importance of considering each person as an active participant in the care process.

Previous studies have shown that sociodemographic characteristics such as age, gender, or educational level and factors related to health history, such as length of stay and type of admission (i.e., planned vs. emergency), influenced the person’s perceptions of individualized care. However, the results were oriented toward quality and outcomes such as satisfaction rather than the process through which care is provided [49–52].

Concerning potential study limitations, it is essential to note that the presented results are specific to the sample and may not be generalizable to broader populations, although conditioned by the lack of other studies using the PCPI-C, as this was the first one.

Additionally, reliance on self-reported instruments introduces the possibility of response bias, wherein participants might offer socially desirable responses or be influenced by individual factors [24]. However, efforts were made to minimize bias and enhance the accuracy of our findings. Confidentiality was strictly maintained throughout the study to encourage genuine participant feedback. All responses were collected anonymously by the researcher, and participants were assured that their data would remain confidential, thus reducing the likelihood of social desirability bias. In addition, potential confounding variables were carefully identified, and the data collection was adjusted to a clinical stable phase of the inpatient stay and closer to discharge.

This study is innovative in several respects, adding to the evidence on PCP in acute care settings [53, 54], to assessments from a multidisciplinary viewpoint of the care received [55, 56], and applying an inventory derived from the PCPF to gather service users’ perceptions. The fact that the PCPF provides a conceptual framework for assessing PCP establishes a solid basis for research and a resource that can be used to trace person-centeredness in different contexts and populations, promoting the comparability of results.

As implications for clinical practice, healthcare providers should receive ongoing training in person-centred care practices, focusing on decision-making, holistic care, and authentic engagement. Additionally, healthcare organizations should support policies promoting person-centred care, ensuring staff have the resources and environment needed to provide high-quality, individualized care. Efforts should also be made to educate and empower patients, encouraging them to take an active role in their healthcare decisions and fostering a collaborative care environment. By addressing these implications, healthcare services can better meet the needs of older adults, improving care quality and patient satisfaction.

## Conclusions

Assessing systematically the perceptions of older people with chronic illness about PCP in acute care from a multidisciplinary perspective based on the PCPF enables us to identify and characterize PCP perceptions in this particular context. *Working with the person’s beliefs and values* was consistently recognized in practice. Nevertheless, *sharing decision-making* and *working holistically* were identified as less present, revealing the need for improvement. Targeted intervention in these areas is required to develop sustainable practices specifically adapted to hospitalized older adults.

The absence of influence of sociodemographic characteristics and health history on the perception of the PCP suggests that in the person-centered process domain, instead of a standard pattern of care shared by the population, we observe a personalized approach to care, with each individual experiencing it uniquely.

The findings of this research enhance the expanding body of evidence supporting the use of the PCPI as a reliable psychometric instrument that enables the recognition of structural concepts from an established theory in practical applications, guiding practice improvements. Moreover, this study can establish a basis for the development of PCP in this context and has the potential to inform healthcare policy and practice, guiding the development of strategies that place the unique needs and perspectives of chronically ill older person at the focus of hospital care. Understanding and analyzing the perceptions of hospitalized older adults can contribute to the implementation of person-centered care and, in turn, to the holistic improvement of health care delivery.

### Electronic supplementary material

Below is the link to the electronic supplementary material.

Supplementary Material 1

Supplementary Material 2

## Data Availability

Data is provided within the supplementary information files.

## Abbreviations

6-CIT: Cognitive Impairment Test
PCP: Person–centered Practice
PCPF: Person–centered Practice Framework
PCPI-C: Person–centered Practice Inventory–Care
PCPI-S: Person–centered Practice Inventory–Staff
