# Supplementary material for: Perspectives of older adults with chronic illness on person-centered practice at an inpatient hospital department: a descriptive study
Source: BMC Geriatr. 2024 Aug 29;24:714. doi: 10.1186/s12877-024-05261-1 (PMC11360336; doi:10.1186/s12877-024-05261-1)
Supplement: Supplementary file 1 — Supplementary Material 1 [file 12877_2024_5261_MOESM1_ESM.pdf]

Appendix A

Appendix A.1. Working with the person's beliefs and values

An analysis of variance (ANOVA) was performed to determine the effect of the variables sex, age, living environment, residence, home care, educational level, number of previous hospitalization episodes, health history, actual diagnosis, length of stay and Barthel index on the construct of *working with the person's beliefs and values* (Table A.3). The analysis of Levene's Test $F(187,4) = .87$, p-value = .66, residual Q-Q chart, and residual histogram (Graph A.1) did not show any apparent violation of the model's assumptions of normality and homoscedasticity.

Table A.3. Results of the ANOVA model of *working with the person's beliefs and values* construct. P-values less than the .05 significance level are highlighted in bold.

Predictor	Sum of Squares	D*f*	Mean Square	*F*	*p-value*	Partial η^2
(Intercept)	299.896	1	299.896	1185.167	.000	.884
Sex	.131	1	.131	.519	.472	.003
Age	.878	5	.176	.694	.629	.022
Living environment	.118	1	.118	.466	.496	.003
Residence	.007	1	.007	.028	.867	.000
Home care	.405	2	.203	.801	.451	.010
Actual diagnosis	1.423	5	.285	1.125	.349	.035
Length of stay	1.317	3	.439	1.735	.162	.032
Number of previous episodes	.102	3	.034	.134	.859	.003
Health history	1.076	7	.154	.607	.749	.027
Barthel index	.493	3	.164	.650	.584	.012
Educational level	1.380	4	.345	1.364	.249	.034
Error	39.474	156	.253			

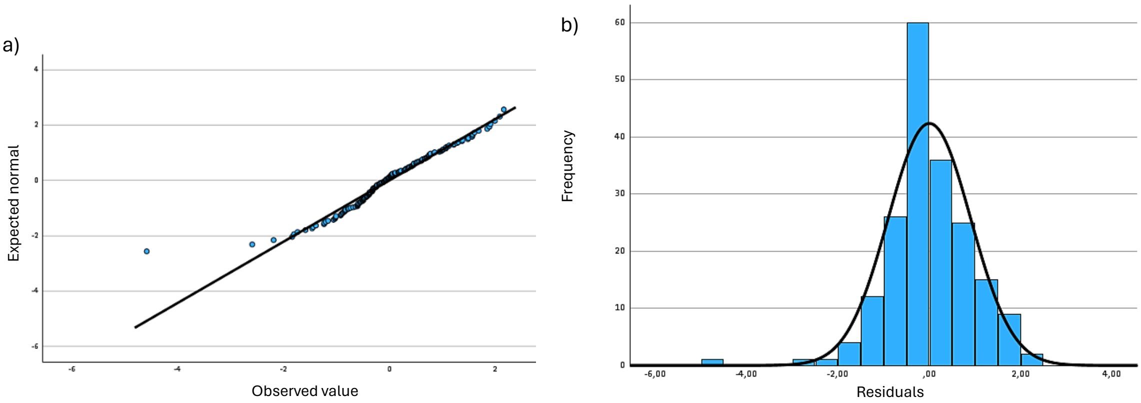

Graph A.1. Q-Q plot of residuals (a) and histogram of residuals (b).

Appendix A.2. Sharing decision-making

An analysis of variance (ANOVA) was performed to determine the effect of the variables sex, age, living environment, residence, home care, educational level, number of previous hospitalization episodes, health history, actual diagnosis, length of stay, and Barthel index on the construct of *sharing decision-making* (Table A.4). The Levene's Test F(187,4) = .77, p-value= .73 and the analysis of the residual Q-Q chart, and residual histogram (Graph A.2) did not show any apparent violation of the model's assumptions of normality and homoscedasticity. Descriptives of the variables with significant effects were determined (Table A.5, Table A.6).

Table A.4. Results of the ANOVA model of *sharing decision-making* construct. P-values less than the .05 significance level are highlighted in bold.

Predictor	Sum of Squares	*Df*	Mean Square	*F*	*p-value*	Partial η^2
(Intercept)	268.297	1	268.297	783.146	.000	.834
Sex	.601	1	.601	1.754	.187	.011
Age	1.087	5	.217	.635	.673	.020
Living environment	.025	1	.025	.072	.788	.000
Residence	.146	1	.146	.426	.515	.003
Home care	.607	2	.303	.885	.415	.011
Actual diagnosis	3.507	5	.701	2.048	.075	.062
Length of stay	4.586	3	1.529	4.463	**.005**	.079
Number of previous episodes	1.665	3	.555	1.620	.187	.030
Health history	1.721	7	.246	.718	.657	.031
Barthel index	.298	3	.099	.290	.832	.006
Educational level	1.220	4	.305	.891	.471	.022
Error	53.437	156	.343			

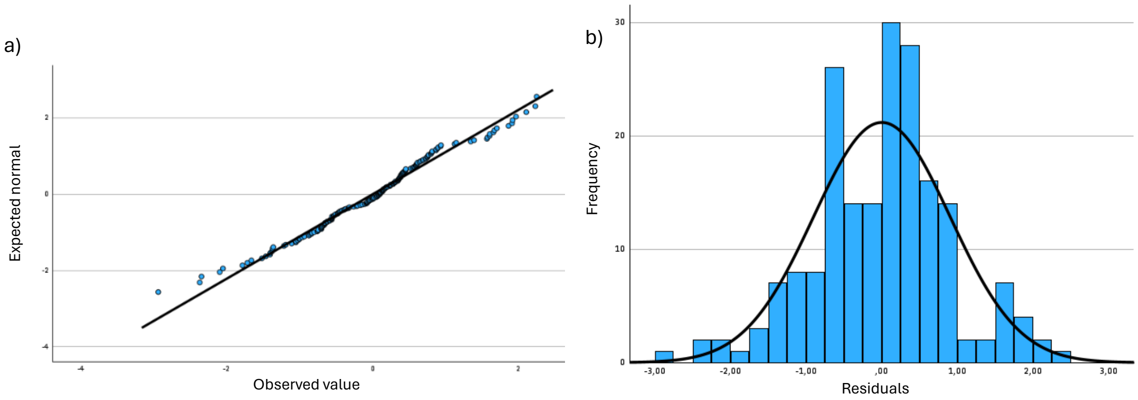

Graph A.2. Q-Q plot of residuals (a) and histogram of residuals (b).

	[1, 3] (N = 6)	[4, 6] (N = 101)	[7, 9] (N = 71)	≥ 10 (N = 14)	Overall (N = 192)
**Sharing decision-making**					
Mean (SD)	4.0 (.16)	3.84 (.58)	3.60 (.64)	4.20 (.41)	3.78 (.60)
Median [Min, Max]	4 [3.75, 4.25]	4 [2.5, 5]	3.75 [1.75, 5]	4 [3.5, 5]	4 [1.75, 5]

Table A.5. Descriptives of *length of stay* in the *sharing decision-making* construct.

	Diseases of the circulatory system (N = 80)	Diseases of the genitourinary system (N = 19)	Diseases of the respiratory system (N = 46)	Haematological and autoimmune diseases (N = 9)	Metabolic, endocrine, and nutritional diseases (N = 12)	Other diseases (N = 26)	Overall (N = 192)
**Sharing decision-making**							
Mean (SD)	3.75 (.64)	3.68 (.61)	3.92 (.50)	3.22 (.68)	3.71 (.61)	3.93 (.49)	3.78 (.60)
Median	4	4	4	3	4	4	4
[Min, Max]	[1.75, 5]	[2.5, 4.5]	[2.5, 5]	[2.5, 4.5]	[2.5, 4.75]	[2.5, 5]	[1.75, 5]

Table A.6. Descriptives of *actual diagnosis* in the *sharing decision-making* construct.

Appendix A.3. Engaging authentically

An analysis of variance (ANOVA) was performed to determine the effect of the variables sex, age, living environment, residence, home care, educational level, number of previous hospitalization episodes, health history, actual diagnosis, length of stay, and Barthel index on the construct of *engaging authentically* (Table A.5). The Levene's Test F(187,4) = .59, p-value = .85 and the analysis of the residual Q-Q chart, and residual histogram (Graph A.3) did not show any apparent violation of the model's assumptions of normality and homoscedasticity.

Table A.7. Results of the ANOVA model of *engaging authentically* construct. P-values less than the .05 significance level are highlighted in bold.

Predictor	Sum of Squares	*Df*	Mean Square	*F*	*p-value*	Partial η^2
(Intercept)	259.539	1	259.539	1084.822	.000	.874
Sex	.019	1	.019	.079	.779	.001
Age	.782	5	.156	.654	.659	.021
Living environment	.044	1	.044	.183	.670	.001
Residence	.100	1	.100	.420	.518	.003
Home care	.671	2	.336	1.403	.249	.018
Actual diagnosis	.539	5	.108	.451	.812	.014
Length of stay	.452	3	.151	.630	.597	.012
Number of previous episodes	.530	3	.177	.738	.531	.014
Health history	.707	7	.101	.422	.888	.019
Barthel index	.316	3	.105	.441	.724	.008
Educational level	.545	4	.136	.570	.685	.014
Error	37.322	156	.239			

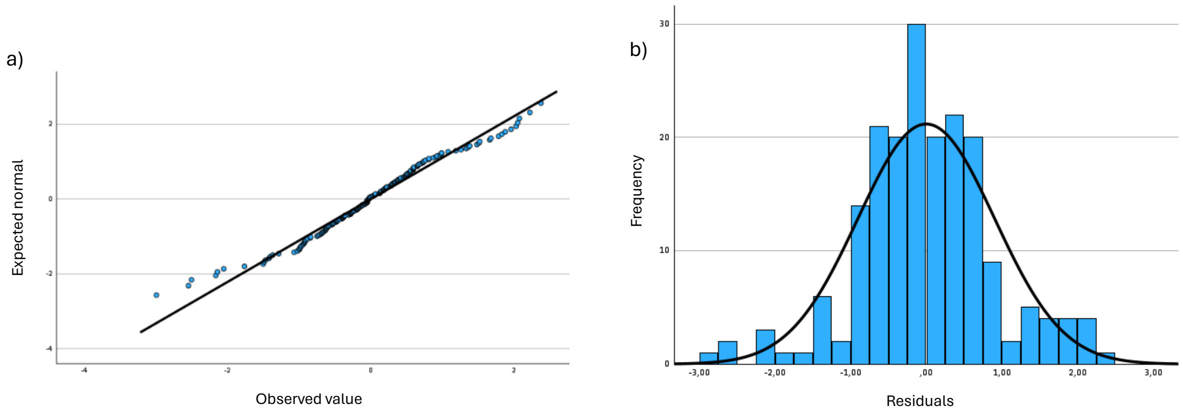

Graph A.3. Q-Q plot of residuals (a) and histogram of residuals (b).

Appendix A.4. Being sympathetically present

An analysis of variance (ANOVA) was performed to determine the effect of the variables sex, age, living environment, residence, home care, educational level, number of previous hospitalization episodes, health history, actual diagnosis, length of stay, and Barthel index on the construct of *being sympathetically present* (Table A.6). The Levene's Test F(187, 4) =

.80, p-value = .71 and the analysis of the residual Q-Q chart, and residual histogram (Graph A.4) did not show any apparent violation of the model's assumptions of normality and homoscedasticity.

Table A.8. Results of the ANOVA model of *being sympathetically present* construct. P-values less than the .05 significance level are highlighted in bold.

**Predictor**	**Sum of Squares**	**D*f***	**Mean Square**	***F***	***p-value***	**Partial** η^2
(Intercept)	283.241	1	283.241	955.168	.000	.860
Sex	.052	1	.052	.175	.676	.001
Age	1.313	5	.263	.886	.492	.028
Living environment	.018	1	.018	.061	.805	.000
Residence	.115	1	.115	.389	.534	.002
Home care	.609	2	.304	1.027	.361	.013
Actual diagnosis	.858	5	.172	.579	.716	.018
Length of stay	1.698	3	.566	1.909	.130	.035
Number of previous episodes	.546	3	.182	.614	.607	.012
Health history	.828	7	.118	.399	.902	.018
Barthel index	.227	3	.076	.255	.858	.005
Educational level	.871	4	.218	.734	.570	.018
Error	46.260	156	.297			

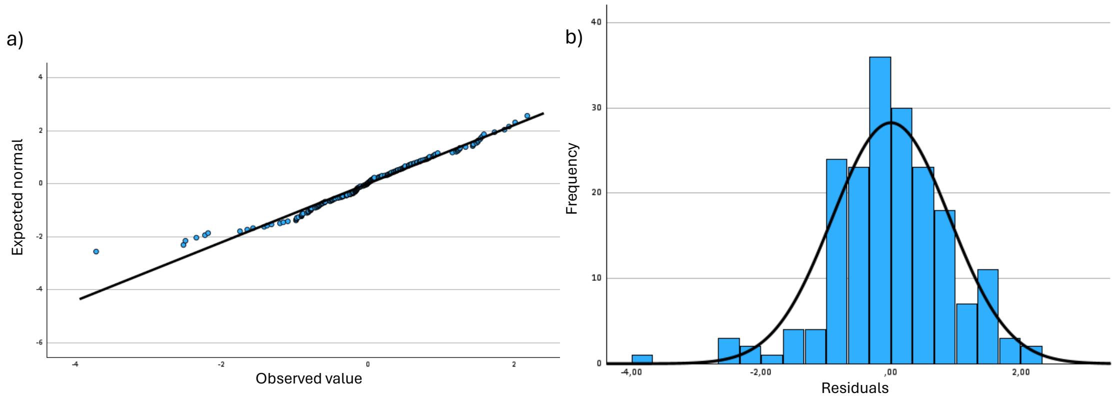

Graph A.4. Q-Q plot of residuals (a) and histogram of residuals (b).

Appendix A.5. Working holistically

An analysis of variance (ANOVA) was performed to evaluate the effect of the variables sex, age, living environment, residence, home care, educational level, number of previous hospitalization episodes, health history, actual diagnosis, length of stay, and Barthel index on the construct of *working holistically* (Table A.7). The Levene's Test F(187,4) = .65, p-value = .81 and the analysis of the residual Q-Q chart, and residual histogram (Graph A.5) did not show any apparent violation of the model's assumptions of normality and homoscedasticity. Descriptives of the variables with significant effect were determined (Table A.10).

Table A.9. Results of the ANOVA model of *working holistically* construct. P-values less than the .05 significance level are highlighted in bold.

**Predictor**	**Sum of Squares**	**D*f***	**Mean Square**	***F***	***p-value***	**Partial** η^2
(Intercept)	257.585	1	257.585	530.126	.000	.779
Sex	.113	1	.113	.232	.631	.001
Age	1.613	5	.323	.664	.651	.021
Living environment	.003	1	.003	.005	.941	.000
Residence	.349	1	.349	.719	.398	.005
Home care	.449	2	.225	.462	.631	.006
Actual diagnosis	3.365	5	.673	1.385	.233	.043
Length of stay	5.167	3	1.722	3.545	**.016**	.064
Number of previous episodes	.696	3	.232	.477	.699	.009
Health history	1.935	7	.276	.569	.780	.025
Barthel index	1.757	3	.586	1.205	.310	.023
Educational level	1.465	4	.366	.754	.557	.019
Error	75.799	156	.486			

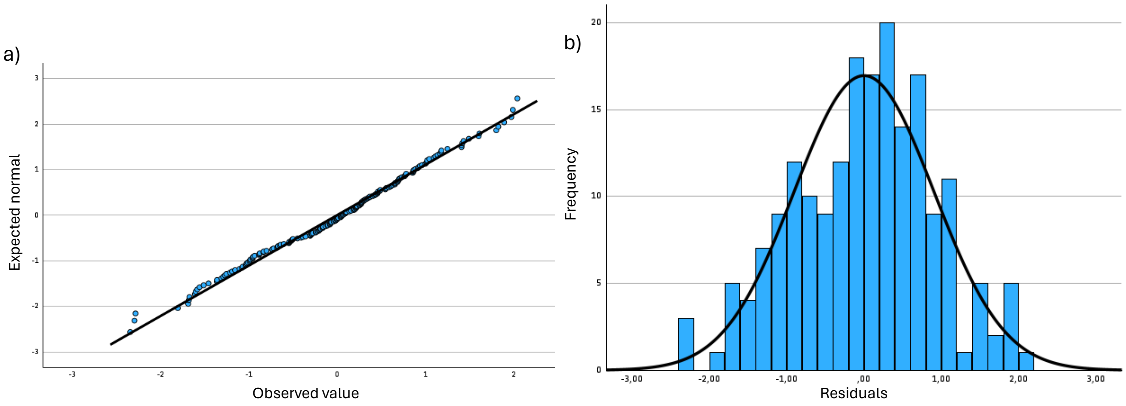

Graph A.5. Q-Q plot of residuals (a) and histogram of residuals (b).

	[1, 3]<br>(N = 6)	[4, 6]<br>(N = 101)	[7, 9]<br>(N = 71)	≥ 10<br>(N = 14)	Overall<br>(N = 192)
**Working holistically**					
Mean (SD)	4.08 (.20)	3.75 (.68)	3.50 (.71)	4.00 (.58)	3.68 (.69)
Median	4.13	4.	3.5	4	4
[Min, Max]	[3.75, 4.25]	[1.75, 5]	[1.75, 4.75]	[3, 5]	[1.75, 5]

Table A.10. Descriptives of *length of stay* in the *working holistically* construct.
