# Supplementary material for: Perspectives of older adults with chronic illness on person-centered practice at an inpatient hospital department: a descriptive study
Source: BMC Geriatr. 2024 Aug 29;24:714. doi: 10.1186/s12877-024-05261-1 (PMC11360336; doi:10.1186/s12877-024-05261-1)
Supplement: Supplementary file 2 — Supplementary Material 2 [file 12877_2024_5261_MOESM2_ESM.pdf]

Appendix B

The correlation between the constructs that make up the PCPI-C was verified (Table B.11). There was a significant correlation in all the constructs, with a p-value > .5. A more robust correlation was revealed between *working with person's beliefs and values* and *being sympathetically present* (p-value = .716), followed by *sharing decision making* and *working holistically* (p-value = .687).

Table B.11. Correlations between the constructs of the PCPI-C

Correlations						
		Working with person's beliefs and values	Sharing decision making	Engaging authentically	Being sympathetically present	Working holistically
Working with person's beliefs and values	Pearson Correlation	1	.612**	.638**	.716**	.538**
	Sig. (2-tailed)		.001	.001	.001	.001
	N		192	192	192	192
Sharing decision making	Pearson Correlation			.582**	.606**	.687**
	Sig. (2-tailed)			.001	.001	.001
	N			192	192	192
Engaging authentically	Pearson Correlation				.598**	.603**
	Sig. (2-tailed)				.001	.001
	N				192	192
Being sympathetically present	Pearson Correlation					.609**
	Sig. (2-tailed)					.001
	N					192
Working holistically	Pearson Correlation					1
	Sig. (2-tailed)					
	N					

**. Correlation is significant at the .01 level (2-tailed).
